# *Alphonsea glandulosa* (Annonaceae), a New Species from Yunnan, China

**DOI:** 10.1371/journal.pone.0170107

**Published:** 2017-02-01

**Authors:** Bine Xue, Yun-Yun Shao, Richard M. K. Saunders, Yun-Hong Tan

**Affiliations:** 1 Key Laboratory of Plant Resources Conservation and Sustainable Utilization, South China Botanical Garden, Chinese Academy of Sciences, Guangzhou, P.R. China; 2 Guangdong Provincial Key Laboratory of Applied Botany, South China Botanical Garden, Chinese Academy of Sciences, Guangzhou, China; 3 University of Chinese Academy of Sciences, Beijing, P.R. China; 4 School of Biological Sciences, The University of Hong Kong, Hong Kong, P.R. China; 5 Center for Integrative Conservation, Xishuangbanna Tropical Botanical Garden, Chinese Academy of Sciences, Mengla, Yunnan, P.R. China; 6 Southeast Asia Biodiversity Research Institute, Chinese Academy of Science, Nay Pyi Taw, Myanmar; Institute of Botany, CHINA

## Abstract

*Alphonsea glandulosa* sp. nov. is described from Yunnan Province in south-west China. It is easily distinguished from all previously described *Alphonsea* species by the possession of glandular tissue at the base of the adaxial surface of the inner petals. Nectar was observed throughout the flowering period, including the pistillate phase and subsequent staminate phase. Small curculionid beetles were observed as floral visitors and are inferred to be effective pollinators since they carry pollen grains. A phylogenetic analysis was conducted to confirm the placement of this new species within *Alphonsea* and the evolution of the inner petal glands and specialized pollinator reward tissues throughout the family.

## Introduction

The genus *Alphonsea* Hook. f. & Thomson (Annonaceae) currently comprises 27 species of shrubs or trees, distributed in wet tropical lowland forests across south and south-east Asia, from India to the Philippines. It is characterized by flowers with saccate petals, ‘miliusoid’ stamens (sensu [1]) and multi-seeded fruits [2]. Keßler [2] recognized 23 species in his taxonomic treatment of the genus, although four additional species were subsequently described from Vietnam [3], Papua New Guinea [4], Borneo [5], and Peninsular Malaysia [6]. Six species have been recorded from China (*A*. *boniana* Finet & Gagnep., *A*. *hainanensis* Merr. & Chun, *A*. *mollis* Dunn, *A*. *monogyna* Merr. & Chun, *A*. *squamosa* Finet & Gagnep., and *A*. *tsangyuanensis* P. T. Li), distributed in Guangdong, Guangxi, Guizhou and Yunnan Provinces [7,8], although *A*. *squamosa* has been treated as a synonym of *A*. *boniana* [2].

Two indigenous trees growing in Xishuangbanna Tropical Botanical Garden and in a small forest patch near Man-zhang Reservoir in Meng-la County, Yunnan Province, were easily identifiable as conspecific and belonging to *Alphonsea*, but could not be assigned to any previously described species. The two trees are readily distinguished from all other *Alphonsea* species as they have multiple flowers (often 5 to 9) in each inflorescence and have glandular tissue at the base of the inner petals. We propose that these two trees represent a hitherto undescribed species, which we describe and name here as *Alphonsea glandulosa*. Molecular phylogenetic analyses based on six chloroplast regions were carried out to ascertain the systematic position of the species.

## Materials and Methods

### Ethics statements

The new species reported in this study was collected from Xishuangbanna Tropical Botanical Garden, Yunnan Province, China, which permitted our field work in the Garden. The other collecting location, Man-zhang Reservoir, Meng-la County, Yunnan, is not a protected area. Since this species is currently undescribed, it is inevitably not currently included in the Chinese Red Data Book [9].

### Phylogenetic analysis

#### Taxon and DNA region sampling

Sequences of 60 species of Annonaceae were downloaded from GenBank, and supplemented with newly generated sequences of the new species, as well as the Chinese species Alphonsea mollis and A. monogyna. The final dataset included 43 accessions representing 31 genera from subfam. Malmeoideae, 15 accessions representing 15 genera from subfam. Annonoideae, four accessions of four genera in subfam. Ambavioideae, and one species of Anaxagorea A. St.-Hil. (subfam. Anaxagoreoideae). Six chloroplast DNA (cpDNA) regions (matK, ndhF, psbA-trnH, rbcL, trnL-F, and ycf1) were amplified for the three species. Genera reported to have inner petal glands were included (see Results and Discussion), with at least one representative species for each. The voucher information and GenBank accession numbers are provided in S1 Table.

#### DNA extraction, amplification and sequencing

Genomic DNA was extracted from herbarium materials using a modified cetyl trimethyl ammonium bromide (CTAB) method [10]. A single amplification protocol was used for amplification of the chloroplast regions, viz.: template denaturation at 94°C for 5 min, followed by 35 cycles of denaturation at 95°C for 30 sec; primer annealing at 50°C for 1 min; and primer extension at 72°C for 1 min, followed by a final extension step at 72°C for 10 min. The primers used were those from [11,12]. PCR products were visualized using agarose gel electrophoresis. Successful amplifications were purified, and sequenced commercially.

#### Alignment and phylogenetic analyses

Sequences were assembled and edited using Geneious ver. 5.4.3 [13] and pre-aligned with the MAFFT [14] plugin in Geneious using the automatic algorithm selection and default settings, with subsequent manual checking and optimization. One inversion of 15 positions in *psbA-trnH* was identified and reverse complemented in the alignment, following a strategy previously applied [15] to retain substitution information in the fragments.

Maximum parsimony (MP) analyses of the six combined regions were conducted using PAUP ver. 4.0b10 [16]. All characters were weighted equally, with gaps treated as missing data. The most parsimonious trees were obtained with heuristic searches of 1,000 replicates of random stepwise sequence addition, tree bisection-reconnection (TBR) branch swapping with no limit to the number of trees saved. Bootstrap support (BS) was calculated following [17], with 10,000 simple stepwise addition replicates with TBR branch swapping and no more than 10 trees saved per replicate.

Bayesian analysis was performed using the NSF Extreme Science & Engineering Discovery Environment (XSEDE) application of MrBayes ver. 3.2.2 [18,19] provided by the CIPRES Science Gateway [20,21]. Six partitions based on DNA region identity were used and the parameters for each locus were allowed to evolve independently using the unlinked setting. MrModeltest ver. 2.3 [22] was used to determine the best-fit likelihood model for each locus and the concatenated matrix using the Akaike Information Criterion: the general time-reversible model with a gamma distribution of substitution rates (GTR+G) was chosen for the *matK*, *psbA-trnH* and *trnL-F* regions; and the GTR+I+G model with a proportion of invariant sites was selected for the *ndhF*, *rbcL* and *ycf1* regions. Two independent Metropolis-coupled Markov chain Monte Carlo (MCMCMC) analyses were run. Each search used three incrementally heated and one cold Markov chain, and was run for 10 million generations and sampled every 1,000th generation. The mean branch length prior was set from the default mean (0.1) to 0.01 (brlenspr = unconstrained: exponential (100.0)) to reduce the likelihood of stochastic entrapment in local tree length optima [23,24]. Convergence was assessed using the standard deviation of split frequencies, with values < 0.01 interpreted as indicating good convergence. The first 25% of samples (2,500 trees) were discarded as burn-in, and the post-burn-in samples summarized as a 50% majority-rule consensus tree.

### Morphological observations

The morphological description of the new species was based on the examination of fresh materials and dried herbarium specimens. Morphological comparisons with other species in *Alphonsea* were based on studies of herbarium specimens (from herbaria HITBC, IBSC, K, KUN, PE and SING; institutional acronyms follow the Index Herbariorum [25]), specimen photographs and a literature survey.

Flowers were fixed in FAA (70% alcohol, formaldehyde and glacial acetic acid in a ratio of 90:5:5) for 24 hrs and subsequently stored in 70% alcohol. Samples examined using scanning electron microscopy were dehydrated using an ethanol series, and critical-point dried with a Leica EM CPD300 Automated Critical Point Dryer (Leica, Wetzlar, Germany). The dried materials were later attached to metal stubs using adhesive carbon tabs, sputter-coated with gold/palladium, and viewed using a JSM-6360LV scanning electron microscope (JEOL, Tokyo, Japan) at 5 kV.

For anatomical observations, the fixed inner petals were dissected and transferred into 70% ethanol, stained with Ehrlich’s hematoxylin, dehydrated in ethanol series, infiltrated with xylene, embedded in paraffin wax and serially sectioned at thickness of 10 μm, using a rotary microtome. Subsequently, mounted slides were examined and photographed under a LEICA DM5500 B microscope equipped with a LEICA DFC550 digital camera.

### Nomenclature

The electronic version of this article in Portable Document Format (PDF) in a work with an ISSN or ISBN will represent a published work according to the International Code of Nomenclature for algae, fungi, and plants, and hence the new names contained in the electronic publication of a PLOS article are effectively published under that Code from the electronic edition alone, so there is no longer any need to provide printed copies [26].

In addition, the new name contained in this work has been submitted to IPNI, from where it will be made available to the Global Names Index. The IPNI LSID can be resolved and the associated information viewed through any standard web browser by appending the LSID contained in this publication to the prefix http://ipni.org/. The online version of this work is archived and available from the following digital repositories: PubMed Central and LOCKSS.

## Results and Discussion

### Phylogenetic analysis

The concatenated alignment of the 63-terminal dataset consisted of 7,399 characters. The MP heuristic search retrieved 24 most parsimonious trees of 3,968 steps (consistency index, CI = 0.66; retention index, RI = 0.70).

**Fig 1:**
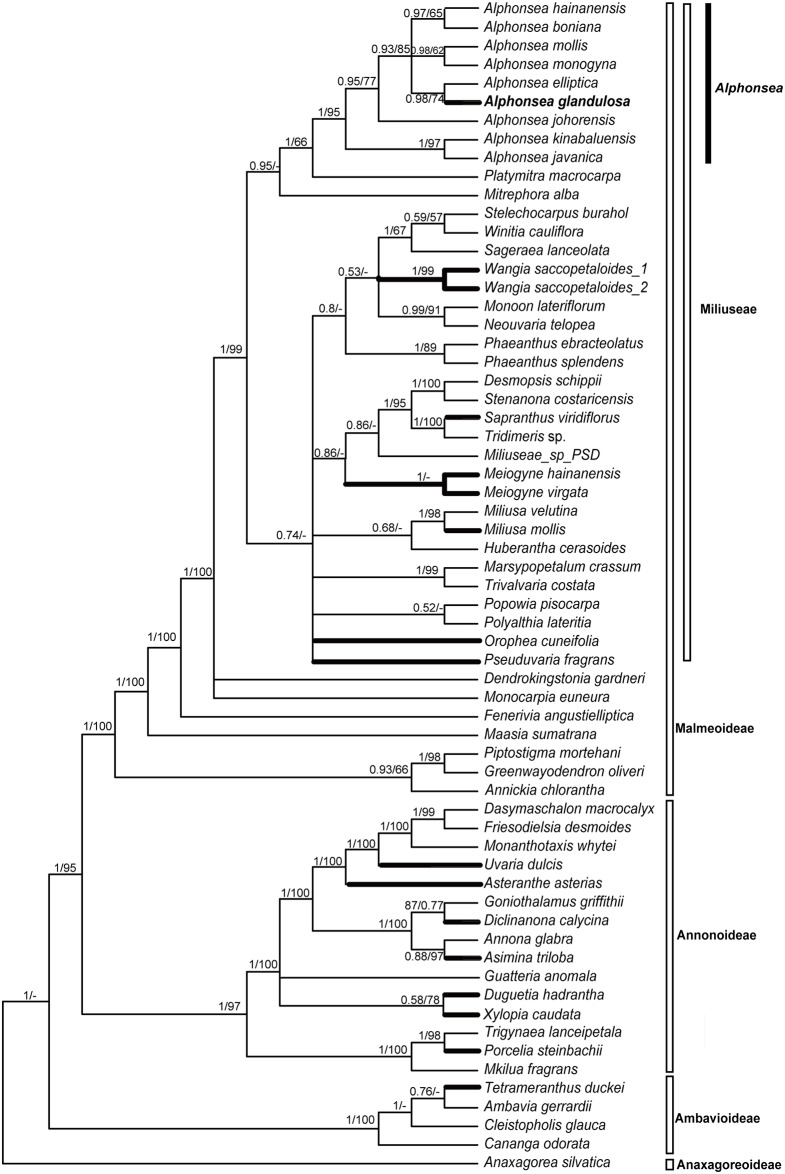
Bayesian 50% majority-rule consensus tree under partitioned models (cpDNA data: *matK*, *ndhF*, *psbA-trnH*, *rbcL*, *trnL-F* and *ycf1*; 63 taxa). Numbers at the nodes indicate Bayesian posterior probabilities and maximum parsimony bootstrap values (> 50%), in that order. Thick lines indicating the clades in which glands or specialized pollinator reward tissues have evolved.

The MP and Bayesian analyses are topologically similar, differing mainly in the relative MP bootstrap (BS) and posterior probability (PP) values for particular groups (Fig 1). The new species, *Alphonsea glandulosa*, is deeply nested within the *Alphonsea* clade and retrieved as sister to *A*. *elliptica*. Although these results confirm that the new species unequivocally belongs to the genus *Alphonsea*, limitations in the extent of taxon sampling within the genus (nine out of 28 species; 32%) preclude any definitive conclusion regarding which species is phylogenetically closest to *A*. *glandulosa*.

### Morphological comparisons

**Fig 2:**
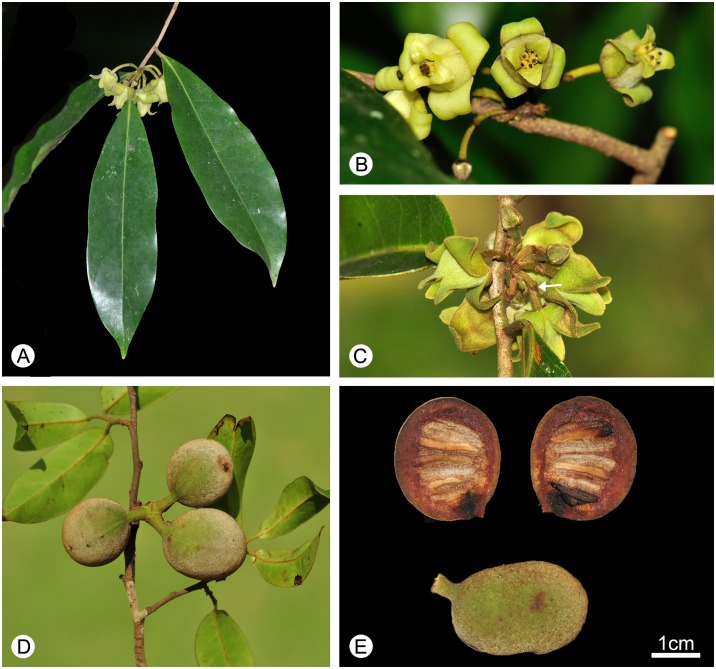
Flower and fruit morphology of *Alphonsea glandulosa*. **A**, Branch, showing leaf-opposed inflorescence position, and lanceolate leaves. **B**, Abaxial view of the inflorescence, showing 5–6 carpels per flower. **C**, Adaxial view of the inflorescence, showing pubescent pedicels with one densely pubescent medial bract (arrowed). **D**, Fruit with subglobose monocarps. **E**, Single monocarp, dissected to show seed arrangement.—Photos: A–E, Yun-hong Tan.

**Fig 3:**
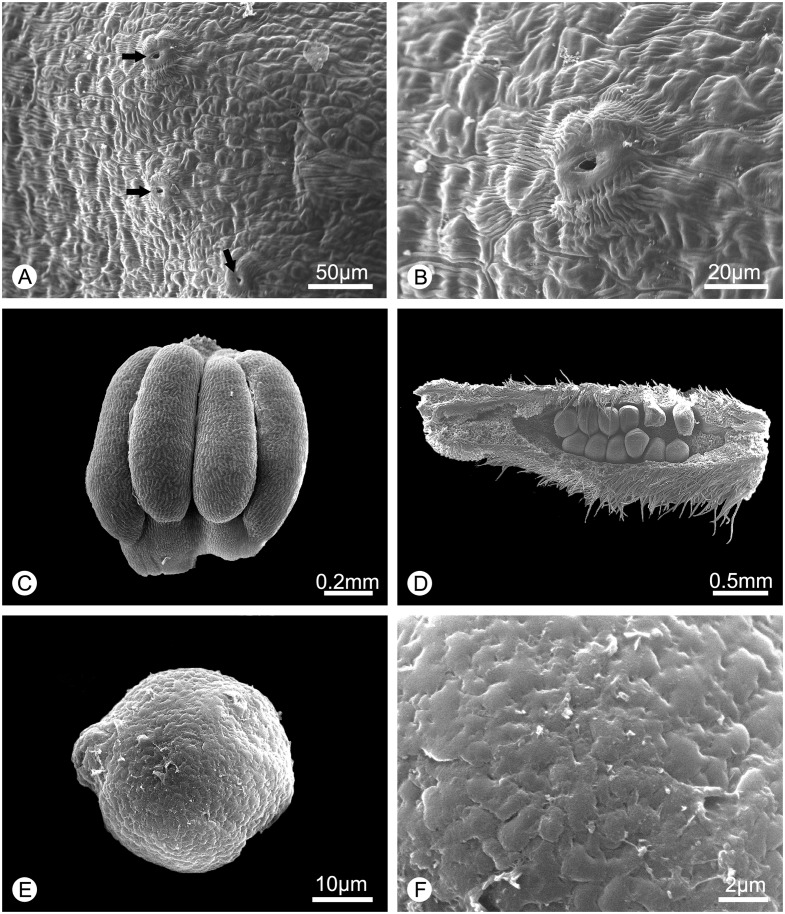
Morphology of the surface of the inner petal glands, stamen, carpel and pollen of *Alphonsea glandulosa* (scanning electron micrographs). **A**, Surface of the glandular tissue, showing the nectary stomata (arrowed). **B**, Close-up of the nectary stomata. **C**, Stamen. **D**, Carpel, dissected to show biseriate ovules. **E**, Pollen grain. **F**, Rugulate pollen exine ornamentation.

*Alphonsea glandulosa* flowers have saccate petals (Fig 2A–2C), ‘miliusoid’ stamens in which the connective does not extend over the thecae (Fig 3C), and hairy ovaries with up to 13 biseriate ovules (Fig 3D). These characters corroborate its placement in the genus *Alphonsea*.

*Alphonsea glandulosa* is unusual in the genus, however, in having multiple flowers (often 5 to 9) in each inflorescence (Fig 2A–2C). Although most *Alphonsea* species have inflorescences with only 1–4 flowers, there are two species, *A*. *philastreana* (Pierre) Finet & Gagnep. and *A*. *ventricosa* (Roxb.) Hook. f. & Thomson, which have more than four flowers per inflorescence [2].

*Alphonsea glandulosa* can easily be distinguished from *A*. *ventricosa*, even from vegetative characters: *A*. *ventricosa* has large leaves (12–27 cm by 4–8 cm) that are distinctly thick and coriaceous, whereas the leaves of the new species are smaller (6–19 cm by 3–5.5 cm) and slightly coriaceous. *A*. *ventricosa* furthermore differs in having flowers with more stamens (40–50, in four whorls) and more carpels (10–12) per flower, and in having fruits with larger monocarps (up to 6 cm long, 4 cm in diameter) and longer stipes (*ca*. 3 cm). *Alphonsea glandulosa* has flowers with 26–35 stamens in three whorls and 4–7 carpels per flower, fruits that are 2–4 cm long and 1.5–3 cm in diameter, and stipes up to 10 mm long.

**Fig 4:**
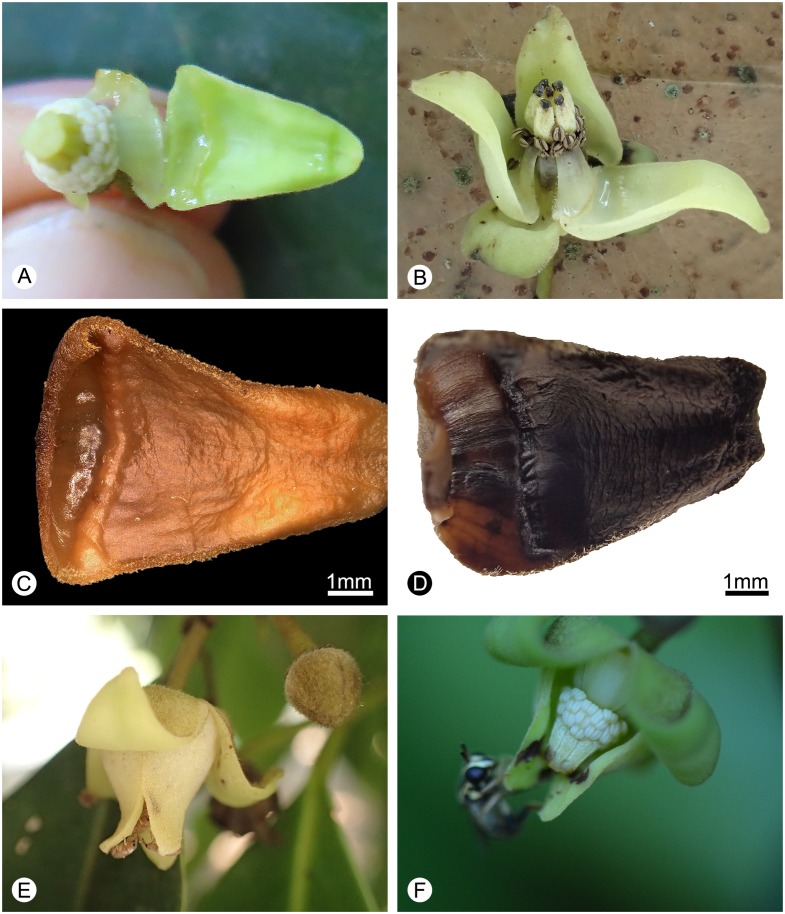
Glandular tissue, nectar and flower visitors of *Alphonsea glandulosa*. **A**, Nectar at pistillate phase. **B**, Nectar at the end of staminate phase. **C**, Morphology of the glandular tissue after FAA fixation. **D**, Morphology of the glandular tissue after rehydration from dried specimens. **E**, Small curculionid beetles observed visiting the flowers. **F**, Bee observed visiting the flower.—Photos: A, C, F, Yun-Yun Shao; B, D, E, Bine Xue.

In contrast, *Alphonsea glandulosa* is vegetatively more similar to *A*. *philastreana*. The two species differ, however, in the number of secondary veins on each side of leaf, the indumentum of the abaxial surface of the flower buds, pedicel length, the number of carpels per flower, and the shape of the stigma. *Alphonsea glandulosa* has 8–18 nerves on each side of the leaf, whereas *A*. *philastreana* has 8–9 [2,27]. The abaxial surface of flower buds of *A*. *glandulosa* is greyish to yellowish pubescent, whereas that of *A*. *philastreana* is densely rusty tomentose [27,28]. Pedicel length and the number of carpels per flower are useful characters for species delimitation: Keβler [2], for example, frequently used these characters in his key to species of *Alphonsea*. Although the name *A*. *philastreana* was validated using a protologue description based on flower buds and hence difficulties were encountered when drawing comparisons, the difference in the length of the pedicel in the two species is nevertheless very significant when buds of a similar size are compared: the pedicel of flower buds that are *ca*. 3–4 mm in diameter, for example, is 10–20 mm long in *A*. *glandulosa* (Fig 2B and 2C) but only *ca*. 3 mm long in *A*. *philastreana* [2,28]. Although previous descriptions of *A*. *philastreana* state that it has (4–)6 carpels per flower and the stigma is globose [27–29], Keβler [2] counted only three carpels in all studied buds and found that the stigma is actually two-headed (deeply bilobed). The two-headed stigma may have misled previous authors into counting six carpels from above. In contrast, *A*. *glandulosa* has between four and seven carpels (Figs 2B and 4B), and the stigma is globose to shallowly bilobed. Ovule numbers of the two species are also different: *A*. *glandulosa* has 10–13 ovules per carpel, whereas *A*. *philastreana* has 14–18(–20) [27–29].

Amongst the *Alphonsea* species sampled in our phylogenetic study, *A*. *elliptica* is retrieved as sister to *A*. *glandulosa* (Fig 1). The differences between these two species are nevertheless clear: the inflorescences of *A*. *glandulosa* are composed of (3–)5–9(–13) flowers, whereas those of *A*. *elliptica* are generally single-flowered (rarely with up to three flowers) [2,30]. *Alphonsea glandulosa* furthermore has stamens in three whorls, whereas *A*. *elliptica* has stamens in four whorls [2].

The most distinctive diagnostic character of *Alphonsea glandulosa*, however, is the possession of nectar glands at the base of the inner petals (Fig 4A–4D). The gland is clearly visible irrespective of preservation technique: in fresh (Fig 4A and 4B) and FAA-fixed (Fig 4C) material the gland is clearly ridge-shaped, and in dried specimens it is apparent as a distinct groove (Fig 4D); it is even obvious in small flower buds.

**Fig 5:**
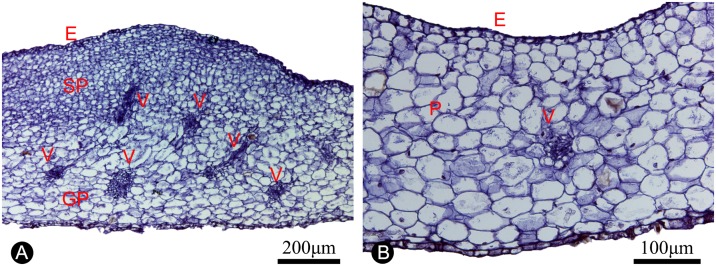
Anatomical structure of the inner petal glands and adjacent non-glandular part of the inner petals (paraffin sections). **A**, Section of the inner petal nectary gland, showing the nectary epidermis (E), secretory parenchyma (SP), ground parenchyma (GP), and vascular tissue (V). **B**, Section of the non-glandular part of the inner petals, showing the epidermis (E), parenchyma (P) and vascular tissue (V).

Anatomically, the nectar glands consist of four distinct tissues (Fig 5A), similar to those described for other species [31–33]: (i) epidermis; (ii) subepidermal secretory parenchyma: several layers of small cells with densely staining cytoplasm; (iii) ground parenchyma: several layers of larger cells, more loosely packed than those of the secretory parenchyma; and (iv) vascular bundles. The anatomical structure is distinct from the non-glandular part of the inner petals (Fig 5B), which only consists of epidermis, several layers of homogeneous parenchyma and a few vascular bundles. The surface of the nectar glands also differs from the surrounding epidermis: nectar stomata, which are raised slightly above the epidermis with an aperture for nectar secretion [32], are found across the surface of the glandular tissue (Fig 3A and 3B), but are otherwise absent from the non-glandular part of the inner petals. The ultrastructure of the glands is similar to those of *Pseuduvaria froggattii* (F.Muell.) Jessup [34, 35].

To conclude, both the molecular and morphological data support the placement of the new species in *Alphonsea*. It differs from all previously described species, and therefore represents a new species.

### Evolution and function of nectar glands

Nectar glands have never previously been recorded in *Alphonsea* [2,36], and it therefore appears that within the genus nectar glands are autapomorphic for *A*. *glandulosa*.

Glandular or specialized pollinator food reward tissues are found in several other genera in the family on different parts of the inner petals, often on the lower part of the adaxial surface, viz. *Asimina* Adans. p.p. [36,37], *Asteranthe* Engl. & Diels [36], *Diclinanona* Diels [36,38], *Duguetia* A.St.-Hil. p.p. [36,39,40], *Meiogyne* Miq. [11,36,41], *Miliusa* Lesch. ex A.DC. p.p. [42–44], *Pseuduvaria* Miq. p.p. [35,36], *Sapranthus* Seem. [36,45], *Tetrameranthus* R.E.Fr. [36,46,47] and *Wangia* X.Guo & R.M.K.Saunders [12]. Paired glands or specialized food tissues are sometimes located along the margins of the inner petals, as found in some *Asimina* p.p. [36], *Diclinanona* p.p. [36,38], *Porcelia* Ruiz & Pav. p.p. [36,48], *Uvaria* L. p.p. (those species previously classified in *Anomianthus* Zoll. and *Ellipeiopsis* R.E.Fr.) [36,49], and *Xylopia* L. p.p. [36]. In *Orophea* Blume p.p. [36,50,51] and *Pseuduvaria* p.p. [35,36] a pair of glands or specialized food tissues are sometimes present underneath the mitre, i.e. on the upper part of the adaxial surface of the inner petals. In *Miliusa* p.p., thickened specialized tissue is found running inside along the adaxial bilateral midline of the inner petals [42–44]. Van Heusden [36] also stated that *Enicosanthum* Becc. species (now included in *Monoon* [52]) possess inner petal glands, although we have not been able to confirm this observation. Nevertheless, most observations of glandular or specialized tissues were based on the examination of herbarium specimens. Field observations of *Asimina* and *Pseuduvaria* spp. confirm the secretion of nectar [34,37,53]. For *Sapranthus* and *Meiogyne* spp., however, no liquid secretions have been observed (George Schatz, *pers*. *comm*); it is therefore debatable whether the specialized tissues in these genera represent true glands that secrete an exudate, and further field observation and anatomical comparisons are required.

Chatrou et al. [54] recognized four subfamilies in their recent subfamilial and tribal classification of the Annonaceae, viz. subfam. Anaxagoreoideae, Ambavioideae, Annonoideae and Malmeoideae (Fig 1). The genera mentioned above with glands or specialized tissues on the inner petals are distributed in all the subfamilies except subfam. Anaxagoreoideae. These genera are classified in up to seven different tribes, indicating that this character is likely to have evolved independently on multiple occasions (Fig 1). A summary of the occurrences of the glands and specialized tissues across the family is provided in Table 1. In the tribe Miliuseae Hook.f. & Thomson, which includes *Alphonsea*, inner petal glands and specialized tissues have evolved independently in seven genera (Table 1; Fig 1).

**Table 1:** The occurrence of inner petal glands and specialized pollinator reward tissues in genera, tribes and subfamilies across the family Annonaceae.

Subfamilies	Tribes	Genera
Ambavioideae	–	*Tetrameranthus*
Annonoideae	tribe Annoneae Endl.	*Asimina* p.p., *Diclinanona*
	tribe Bocageeae Endl.	*Porcelia* p.p.
	tribe Duguetieae Chatrou & R.M.K.Saunders	*Duguetia* p.p.
	tribe Monodoreae Baill.	*Asteranthe*
	tribe Uvarieae Hook.f. & Thomson	*Uvaria* p.p.
	tribe Xylopieae Endl	*Xylopia* p.p.
*Malmeoideae*	tribe Miliuseae Hook.f. & Thomson	*Alphonsea* p.p., *Meiogyne*, *Orophea*, *Pseuduvaria* p.p., *Sapranthus*, *Wangia*, *Miliusa* p.p.

Pollination ecology studies have been undertaken for several species that possess glandular or specialized inner petal tissues, viz.: *Asimina obovata* (Willd.) Nash and *A*. *pygmaea* (W. Bartram) Dunal, which are pollinated by large scarabaeid beetles [37]; *Pseuduvaria froggattii*, pollinated by Drosophilidae and other flies [34]; *Pseuduvaria mulgraveana* Jessup, pollinated by small diurnal nitidulid beetles [53]; and *Sapranthus palanga* R.E.Fr., pollinated by tenebrionid, nitidulid, and scarabaeid beetles and apid bees [45]. In *Asimina obovata* and *A*. *pygmaea*, beetles were observed to consume the corrugated inner petal tissues that were sometimes also observed to secrete a small volume of exudate [36]. In *Pseuduvaria froggattii* and *P*. *mulgraveana*, the secretion of nectar from the inner petal glands was recorded in detail and shown to be concomitant with the anthetic changes: nectar was present throughout the pistillate and staminate phases in hermaphroditic flowers of both species, and in staminate flowers of *P*. *mulgraveana* [34,53]. Pollinators were observed consuming the nectar [34,53], indicating that the nectar glands function as a food reward for pollinators throughout anthesis.

As with all hermaphroditic-flowered Annonaceae species [55], *Alphonsea glandulosa* is protogynous. In *A*. *glandulosa*, abundant sweet nectar was observed to be secreted from the beginning of the pistillate phase until the end of the staminate phase (Fig 4A and 4B). Around three to eight small curculionid beetles were found inside the flowers (Fig 4E), and pollen was observed on their bodies, indicating that they are likely to be effective pollinators. The nectar is likely to provide a food reward to those beetles, as the beetles were observed to consume it. One opportunist bee was also observed visiting the flower (Fig 4F), although it is unlikely that the bee could effectively pollinate the flower because of the presence of the floral chamber formed by the inner petals, which would restrict access by the bees [56].

### Taxonomic treatment

**Fig 6:**
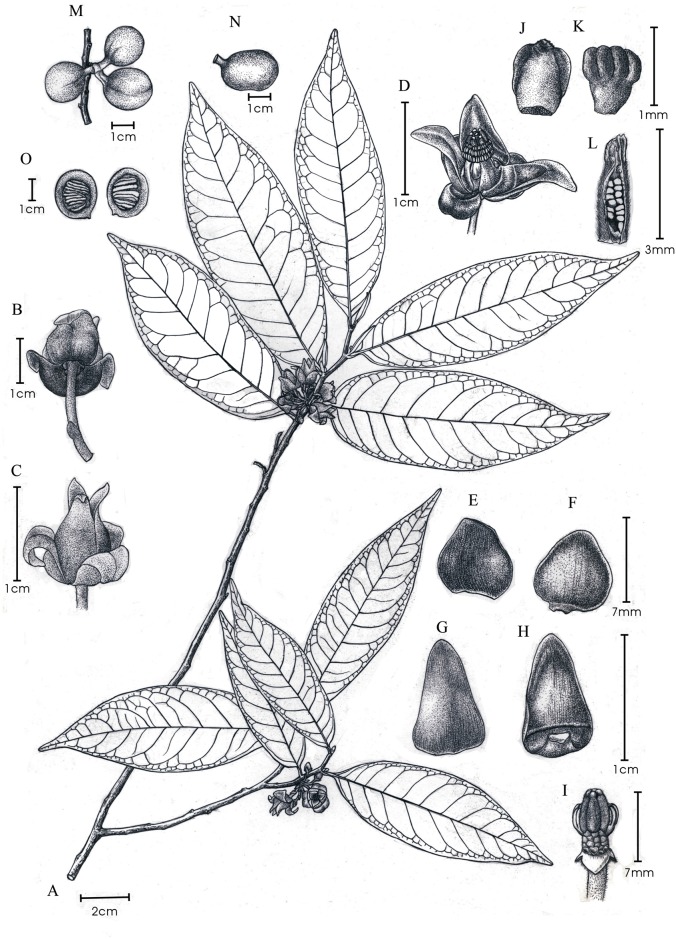
*Alphonsea glandulosa* sp. nov. **A**, Flowering branch. **B–D**, Flower (B, Lower view. C, Lateral view. D, Top view, showing six carpels and three rows of stamens). **E–F**, Outer petal (E, Abaxial view. F, Adaxial view). **G–H**, Inner petal (G, Abaxial view. H, Adaxial view, with nectar glands at the base). **I**, Flower with petals removed, showing the attachment of stamens and carpels. **J–K**, Stamen (J, Adaxial view. K, Abaxial view). **L**. Carpel, longitudinal section, showing ovule arrangement. **M**, Fruit, composed of separate monocarps. **N**, Single monocarp. **O**, Longitudinal section of monocarp, showing seed arrangement. Drawn by Yun-xiao Liu.

***Alphonsea glandulosa*** Y.H. Tan & B. Xue, ***sp*. *nov***. [urn:lsid:ipni.org:names:77159533–1] (Figs 2, 3, 4, 5 and 6)

Type: CHINA. Yunnan Province, Meng-la County, Meng-lun, Man-zhang Reservior, 21°55′53″N, 101°10′58″E, alt. 625 m. *Y*. *H*. *Tan 10145*, 2016.04.07 (fl.) (holotype HITBC; isotypes IBSC, KUN)

#### Diagnosis

*Alphonsea glandulosa* is unique amongst *Alphonsea* species in having a nectar gland at the base of the adaxial surface of each inner petal. It is most similar to *A*. *philastreana* (Pierre) Finet & Gagnep., but differs in having a greater number of secondary veins on each side of the leaf, greyish to yellowish pubescent flower buds, longer pedicels, a greater number of carpels per flower, a smaller number of ovules per carpel, and globose to shallowly bilobed stigmas.

#### Description

Trees to 15–20 m tall, *ca*. 25–30 cm dbh. Bark brownish, fissured. Young twigs green, puberulent, soon become greyish and glabrous. Petioles 3–8 mm long, 1–2 mm in diameter, transversely densely striate; leaf laminae narrlowly elliptic, elliptic or ovate, 6–19 × 3–6.7 cm, base cuneate, apex acuminate, slightly coriaceous, abaxially sparsely pubescent to glabrescent, adaxially glabrous; midrib impressed and glabrous above, raised and hairy to glabrous below; secondary veins 8–18 on each side of the leaf, parallel, diverging at 45–60° from midrib, anastomosing within margin, distinctly raised below; tertiary veins reticulate, prominent abaxially. Inflorescences leaf-opposed or supra-axillary; (3–)5–9(–13) flowers per inflorescence (Fig 2A–2C). Peduncles absent or up to 3 mm long (Fig 2A–2C). Pedicels 10–20 mm long, 1–1.5 mm in diameter, pubescent, with one densely pubescent median bract (Fig 2A–2C). Sepals ovate, 1.5–2 × 1.5–2.5 mm, hairy abaxially, glabrous adaxially; outer petals ovate, 10–14 × 6–8.5 mm, base acute, apex acute, tomentose abaxially, sparsely hairy to glabrous adaxially; inner petals narrower, 10–14 × 5–8 mm wide, hairy abaxially, glabrous adaxially, with glandular tissue near the base (Fig 4A–4D), apparent as ridge in fresh (Fig 4A and 4B) and FAA-fixed (Fig 4C) material, and as distinct groove in dried specimens (Fig 4D). Stamens ‘miliusoid’ with very short connective prolongation not extending over pollen sacs, 26–35 per flower, *ca*. 1 mm long, in 3 whorls (Figs 3C, 4A and 4F). Carpels 4–7 per flower, *ca*. 3 mm long, with short brown hairs (Figs 2B and 4B); ovules 10–13 per carpel, biseriate (Fig 3D). Fruiting pedicels 7–20 mm long, 3 mm in diameter; monocarps 1–7 per fruit, subglobose to cylindrical, *ca*. 2–4 cm long, 1.5–3 cm in diameter, yellow when mature, greyish pubescent, smooth, sometimes with a longitudinal ridge or groove, apex rounded; stipe to 10 mm long, *ca*. 4 mm thick (Fig 2D and 2E). Seeds flattened-ellipsoid, up to 13 per monocarp (Fig 2E). Pollen grains solitary, subspherical, 30–40 μm in diameter, rugulate (Fig 3E and 3F).

#### Etymology

The specific epithet reflects the presence of nectar glands at the base of the adaxial surface of each inner petal.

#### Additional specimens examined (paratypes)

China. Meng-la, Yunnan, 2009-03-25, *Chun-fen Xiao C100647* (HITBC); 2015-04-29, *B*. *Xue 188* (IBSC); 2016-04-26, *B*. *Xue 265* (IBSC, KUN); 2016-04-27, *B*. *Xue 268* (IBSC, SING).

#### Distribution

**Fig 7:**
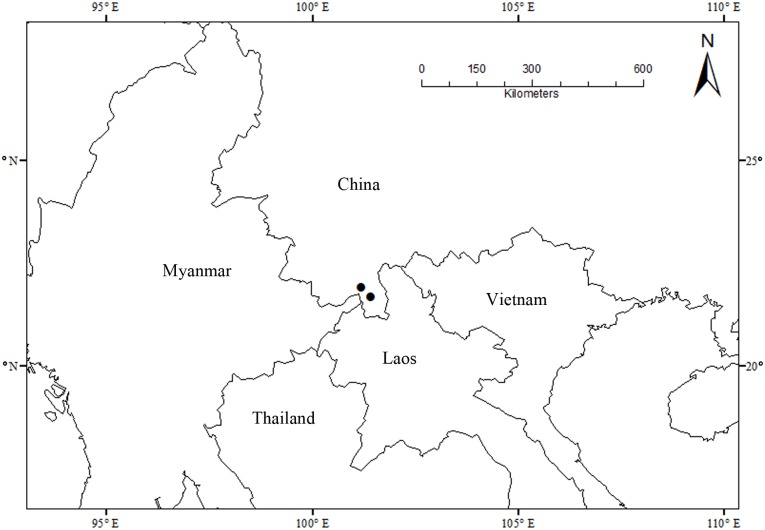
Distribution of *Alphonsea glandulosa*.

Only known from two localities in Yunnan, China (Fig 7).

#### Ecology and phenology

In evergreen forests. Flowering specimens collected in March to May, and fruiting specimens in June to July.

#### IUCN Conservation Status

Only two individuals were found in Meng-la County, Yunnan Province. The primary forests in Xishuangbanna have been under severe pressure from agricultural expansion over the last 30 years, and below 900 m elevation most unprotected forest has been replaced by rubber plantations [57]. The tree growing in the forests close to Man-zhang Reservoir in Meng-la County is located at the edge of a rubber plantation. One of the authors, Yun-Hong Tan, has undertaken an extensive field survey in Xishuangbanna, but was unable to locate other individuals. Perhaps because of the dearth of individuals, the level of fruitset in the two trees is poor. On the basis of current IUCN red list categories and criteria [58], we therefore recommend critically endangered status, CR D.

## Supporting Information

S1 Table GenBank accession numbers and voucher information for the materials used in this study. (XLSX)
